# Emetine induces chemosensitivity and reduces clonogenicity of acute myeloid leukemia cells

**DOI:** 10.18632/oncotarget.8096

**Published:** 2016-03-15

**Authors:** Josep Maria Cornet-Masana, Daniel Moreno-Martínez, María Carmen Lara-Castillo, Meritxell Nomdedeu, Amaia Etxabe, Niccolò Tesi, Marta Pratcorona, Jordi Esteve, Ruth M. Risueño

**Affiliations:** ^1^ Josep Carreras Leukaemia Research Institute, Barcelona, Spain; ^2^ Department of Hematology, Hospital Clínic, Institut d'Investigacions Biomèdiques August Pi i Sunyer (IDIBAPS), Barcelona, Spain

**Keywords:** AML, emetine, anti-leukemia drug

## Abstract

Acute myeloid leukemia (AML) is an hematologic neoplasia characterized by the accumulation of transformed immature myeloid cells in bone marrow. Although the response rate to induction therapy is high, survival rate 5-year after diagnosis is still low, highlighting the necessity of new novel agents. To identify agents with the capability to abolish the self-renewal capacity of AML blasts, an *in silico* screening was performed to search for small molecules that induce terminal differentiation. Emetine, a hit compound, was validated for its anti-leukemic effect *in vitro*, *ex vivo* and *in vivo*. Emetine, a second-line anti-protozoa drug, differentially reduced cell viability and clonogenic capacity of AML primary patient samples, sparing healthy blood cells. Emetine treatment markedly reduced AML burden in bone marrow of xenotransplanted mice and decreased self-renewal capacity of the remaining engrafted AML cells. Emetine also synergized with commonly used chemotherapeutic agents such as ara-C. At a molecular level, emetine treatment was followed by a reduction in HIF-1α protein levels. This study validated the anti-leukemiceffect of emetine in AML cell lines, a group of diverse AML primary samples, and in a human AML-transplanted murine model, sparing healthy blood cells. The selective anti-leukemic effect of emetine together with the safety of the dose range required to exert this effect support the development of this agent in clinical practice.

## INTRODUCTION

Although the outcome of therapy for leukemia has improved over the years, less than a third of adults with acute myeloid leukemia (AML) are cured by current treatment, a fact strengthening the need for new therapeutic approaches [1, 2]. The discovery of the leukemia stem cells (LSC) as a subpopulation of leukemic blasts with stem cell-like properties, such as self-renewal and differentiation capacity [3, 4], established the notion that the emergence of drug resistance and clinical relapse following initial remission is related to the persistence of chemotherapy-resistant LSCs [5]. Therefore, eradication of LSCs is considered necessary for the definitive cure of the disease.

Chemotherapy currently used for AML is composed by the combination of conventional antineoplastic agents with considerable myelotoxicity and extramedullary side effects that may be irreversible and limit their therapeutic potential [6]. On the other hand, the knowledge accumulated over the past decades on the nature of leukemic cells indicates that such cells can be converted into non-dividing, growth arrested cells, with a decreased sensitivity to standard chemotherapy [7]. In this regard, it has been established that differentiation of LSCs leads to an inhibition of self-renewal and, accordingly, a loss of their leukemia initiation capacity [8, 9]. Thus, differentiation therapy constitutes a promising new approach for AML.

Using the gene expression signature associated with vitamin D3-induced differentiation of AML cells, we have identified Emetine as a potential anti-leukemia agent that differentially reduces cell viability and the clonogenic capacity of AML cells sparing healthy blood cells [8].

## RESULTS

The naturally active form of vitamin D3, 1α,25-(OH)2 D3, induces differentiation of normal and leukemic myeloid cells along the monocyte/macrophage lineage [10]. This compound has a dual effect on AML cells: inhibits cell proliferation and, simultaneously, is a very potent inducer of cell differentiation [10]. Using public available repositories, the gene signature associated with vitamin D3-induced differentiation on HL-60 cell line was obtained (Supplementary Tables 1 and 2) and established as the therapeutic target. Using the Connectivity Maps [11], an *in silico* screening was performed to seek for FDA-approved small bioactive compounds that induce a similar gene expression pattern than our gene signature. Emetine, a natural product alkaloid obtained from *Cephaelis ipecacuanha*, displayed the highest score (Supplementary Figure 2).

**Figure 1:**
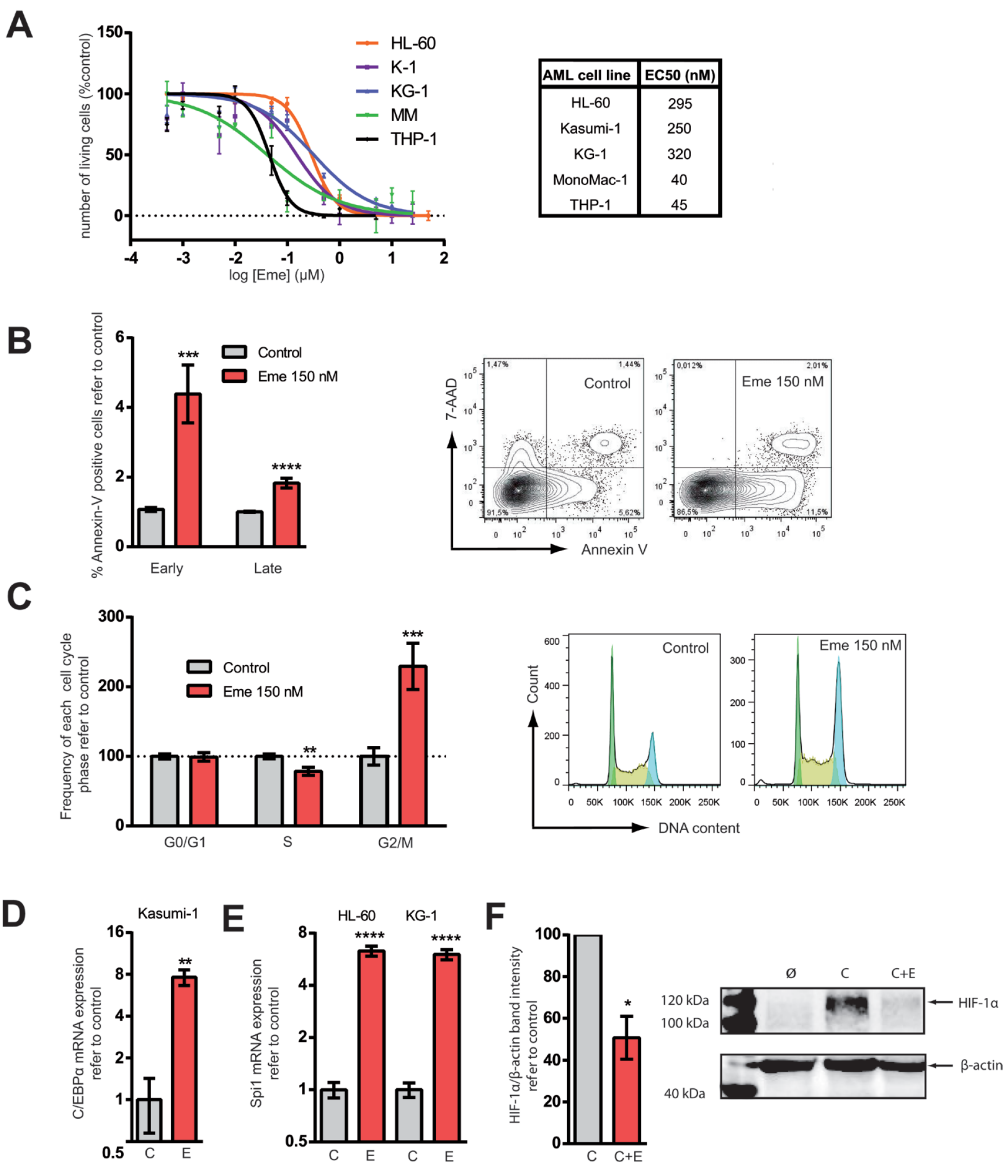
Emetine treatment reduced cell viability, induced apoptosis, prompted AML cells towards differentiation and downregulated HIF-1α **A.** HL-60, KG-1, MonoMac-1 (MM), Kasumi-1 (K-1) and THP-1 AML cell lines were treated for 24 h at different concentrations of emetine (μM). Each point represents the mean value of a biological triplicate and error bars represent SEM. Y-axis: number of live cells relative to vehicle-treated control as assessed by flow cytometry (correct FSC-SSC profile and 7-AAD^−^Hoechst^low^). The EC50 is indicated in the nM range. **B.** MonoMac-1 and Kasumi-1 cell lines were treated with 150 nM emetine (red) or with vehicle control (light grey) for 24 h. Annexin-V staining was measured by flow cytometry. Data from both cell lines are presented combined (*n* = 6 for each line) (left panel). Y-axis: relative frequency of early and late apoptotic cells. Early apoptotic: Annexin V^+^, 7-AAD^−^; late apoptotic: Annexin V^+^, 7-AAD^+^. Representative flow cytometry plot of vehicle-treated or emetine-treated MM (right panel). **C.** HL-60, KG-1, MonoMac-1 and Kasumi-1 cells were treated with 150 nM emetine (red) for 24 h or vehicle control (light grey) and cell cycle was analyzed by flow cytometry. Relative frequency of G0/G1, S and G2/M phases is presented (left panel). Bars represent the mean value of all AML cell lines (*n* = 6 for each cell line) and error bars represent SEM. Representative DNA content flow profile of control and emetine-treated KG-1 cells (right panel). Green represents G0/G1 phase; yellow, S-phase; blue, G2/M. HL-60, KG-1 and Kasumi-1 cells were treated with 150 nM emetine (E, red) for 24 h or with vehicle control (C, light grey). **D.** and **E.** C/EBPα (K-1) and PU.1 (HL-60, KG-1) mRNA levels are represented in K-1 and HL-60 and KG-1 cell lines upon treatment with emetine (E) compared to control (C). **F**. HL-60 and KG-1 cells were treated for 24 h with 50 μM CoCl_2_ (C), 50 μM CoCl_2_ plus 150 nM emetine (C+E) and vehicle control (Ø) [34]. HIF-1α was detected by Western Blot. Left panel represents HIF-1α protein level normalized to β-actin and refer to CoCl_2_ control as assessed by band densitometry. Data from all 5 cell lines are presented combined. Error bar represents SEM. Right panel shows a representative Western blot of HL-60 samples. * *p* < 0.05; ** *p* < 0.01; *** *p* < 0.001; **** *p* < 0.0001.

In order to study the cytotoxic effect of emetine on AML cells, a panel of 5 AML cell lines was treated for 24 h. The EC50 obtained among these cell lines ranged between 40 to 320 nM (Figure 1A). A concentration of 150 nM was chosen as the working EC50 for all cell lines tested. A reduction of the cell viability was observed upon Emetine treatment in a dose-response manner (Figure 1A) together with the induction of apoptosis, as observed by the expression of Annexin-V (Figure 1B). The cell cycle regulation in the presence of emetine was also investigated and a G2/M arrest was observed as shown in Figure 1C. The accumulation of cells in G2/M phase was accompanied in parallel by a reduction of cells in S phase. Thus, emetine reduced cell viability of AML cells by inducing apoptosis and G2/M arrest.

The *in silico* screening was aimed to find drugs that were able to induce differentiation together with cell death. As described previously [12], C/EBPα is inhibited by the fusion protein RUNX1/CBF2T1 that results in the block of granulocytic differentiation observed in this subtype of AML. In Kasumi-1, a RUNX1/CBF2T1 positive AML cell line, C/EBPα mRNA expression was markedly induced after emetine treatment (Figure 1D). Thus, the block in myeloid differentiation is overcome upon treatment with emetine. Similarly to C/EBPα, Spi1 (PU.1) transcriptional factor regulates macrophage differentiation in myeloid progenitors [13]. In the presence of emetine, Spi1 was upregulated similarly to C/EBPα (Figure 1E). Due to the massive cell death detected upon treatment at 24 h (Figure 1A), the evaluation of morphologic changes associated with myeloid differentiation and/or myeloid-differentiation associated cell surface marker expression was challenging. Therefore, emetine not only reduced cell viability of AML cells by inducing apoptosis but also, at least partially, activated the myeloid differentiation transcriptional program as predicted in the *in silico* screening.

Emetine was originally described as a ribosomal-mediated protein [14] and DNA synthesis [15] inhibitor associated to a blocking at the early S phase of DNA replication [16]. In our system, very small differences in the total amount of proteins in the cytoplasm or the protein profile between vehicle- and emetine-treated AML cells were observed (Supplementary Figure 3). The discrepancies in the effect of emetine on the protein profile between previous descriptions and the current analysis can be explained by the smaller doses of this agent used in the current study, as previous works studied emetine mechanism using μM concentrations [14–16]. Conversely, at the nanoMolar range, emetine has been described to inhibit both the activation of HIF-1α by hypoxia and iron chelator-induced HIF-1 activation [17]. AML cells were cultured in hypoxia-like conditions and treated with emetine. As shown in Figure 1F, HIF-1α protein level decreased upon treatment in all AML cell lines tested.

**Figure 2:**
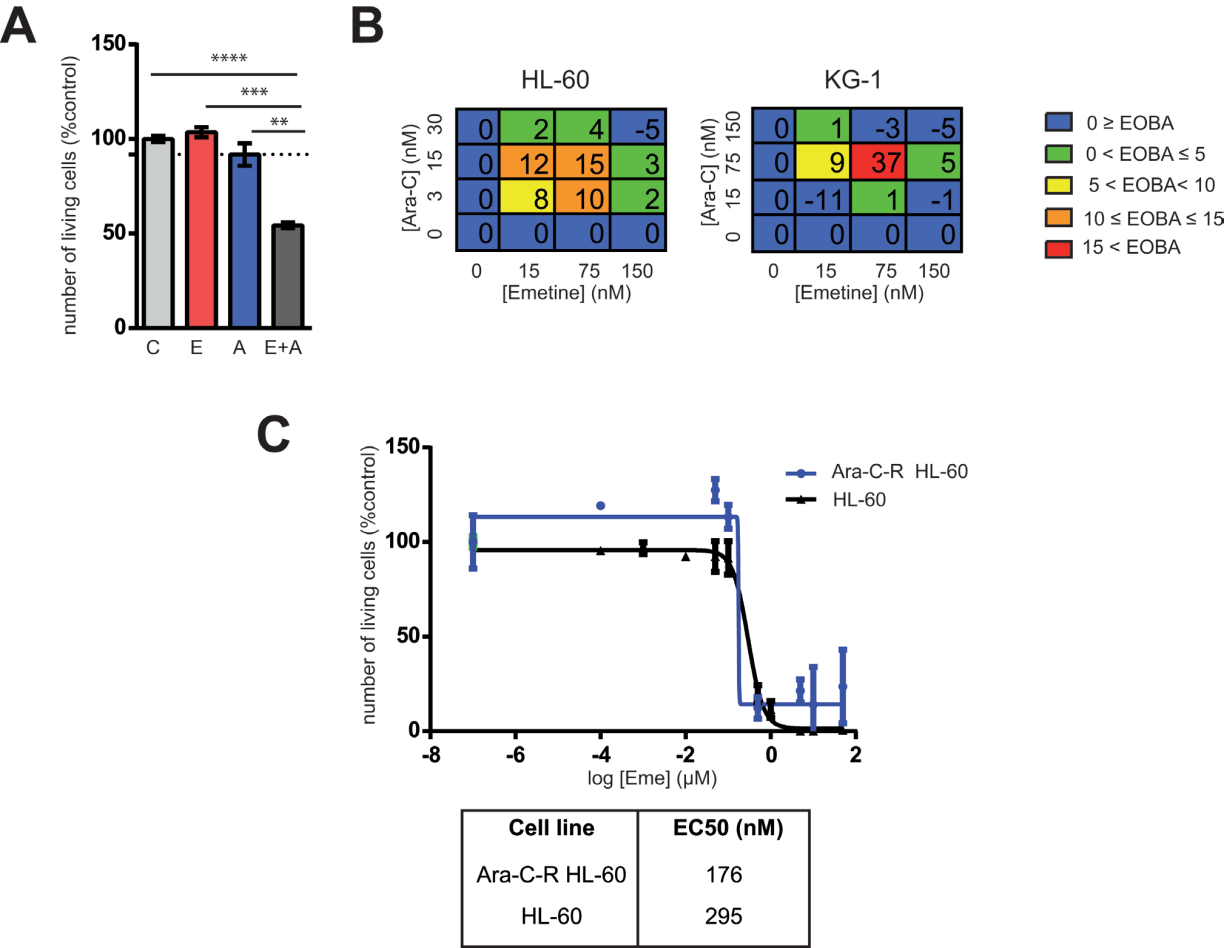
Emetine synergized with ara-C cytotoxicity in AML cells **A.** KG-1 cells were treated with 75 nM Emetine (E, red), 30 nM ara-C (A, blue) or a combination of both (E+A, dark grey). Each point represents a triplicate and error bars represent SEM. Y-axis: relative number of live cells refers to vehicle-treated samples assessed by flow cytometry (7-AAD^−^, Hoechst^low^). Dotted lines represent the expected effect if drugs were additive (as predicted by Bliss additivity). **B.** HL-60 and KG-1 cells were treated with emetine in combination with ara-C at different concentrations for 24 h and cell viability was analyzed by flow cytometry. Tables show color-coded Excess Over Bliss Additivity (EOBA) for each combination assayed expressed as a percentage. Results are a mean of a triplicate. **C.** HL-60 and ara-C-resistant HL-60 (Ara-C-R) AML cell lines were treated for 24 h with increasing concentrations of emetine. Each point represents a triplicate and error bars represent SEM. Y-axis: number of live cells relative to untreated control as assessed by flow cytometry (7-AAD^−^, Hoechst^low^)*p < 0.05; ** *p* < 0.005; ***p < 0.0005.

Combination chemotherapy has been the standard of care in cancer treatment since it is a multitargeted strategy that might result in an increase of both response and tolerability. Moreover, combination chemotherapy might prevent the emergence of treatment-related mutations. Currently, for most AML patients, frontline treatment regimen still involves high doses of chemotherapeutics such as the cell cycle-specific inhibitor cytarabine (ara-C) in combination with a cell cycle-unspecific inhibitor anthracycline such as daunorubicin or idarubicin [1]. In order to determine the combinational effect of emetine with currently used chemotherapeutic agents, AML cells were treated with increasing doses of emetine combined with ara-C. Although no effect on cell viability was observed when 1/10 of the EC50 of either emetine or ara-C was used, a synergistic reduction in cell number equivalent to the EC50 was obtained when both drugs were used simultaneously (Figure 2A). In fact, emetine synergized with ara-C as defined by an excess over Bliss additivism (EOBA [18]) (Figure 2B). Higher EOBA values indicate greater synergy with the drug combination. Interestingly, the sensitivity to emetine treatment as measured by the EC50 is similar in ara-C-resistant HL-60 clones as compared to parental sensitive HL-60 (Figure 2C). Taken together, emetine synergized with currently available chemotherapeutics used for AML treatment.

**Figure 3:**
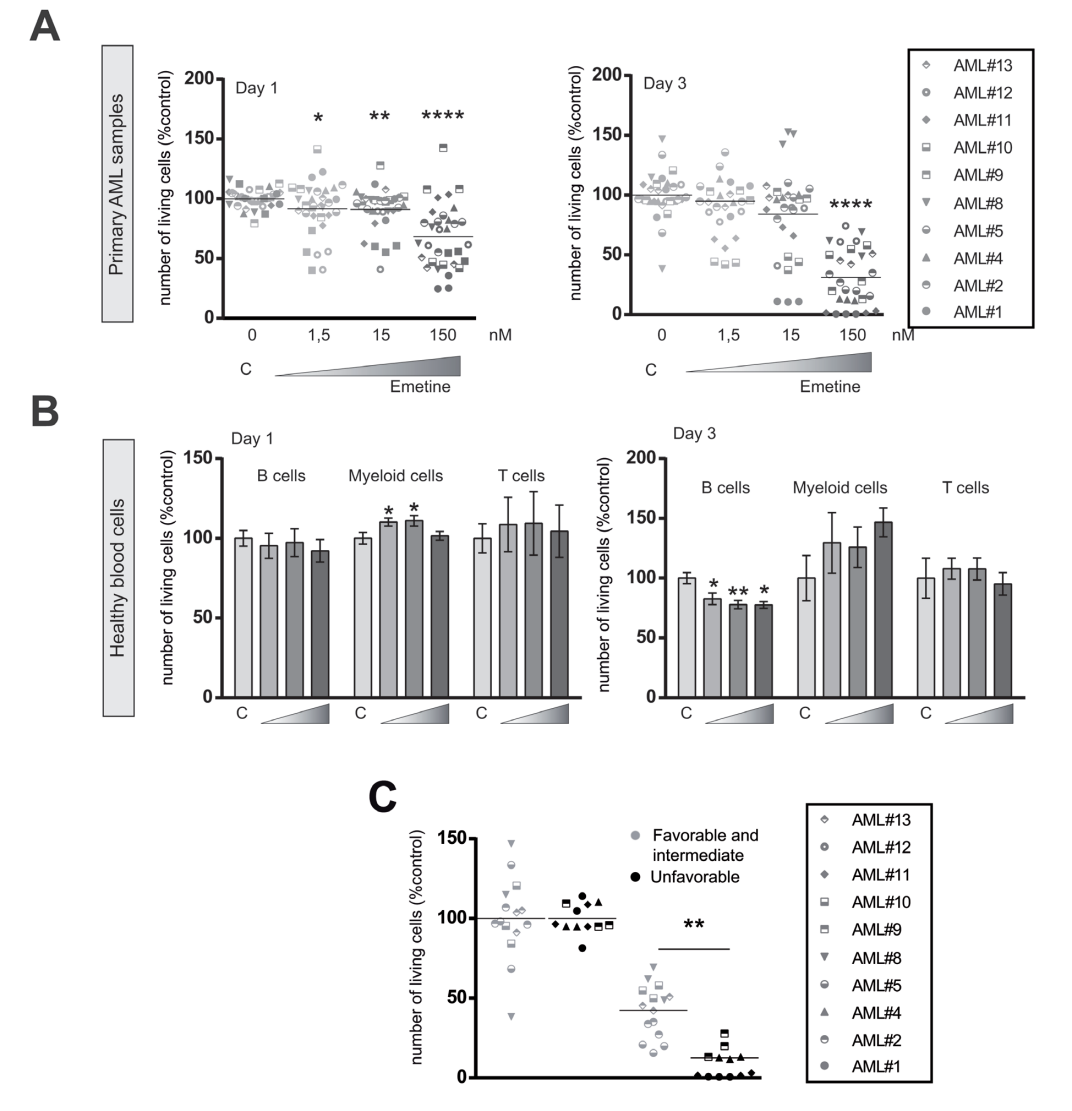
Emetine induced cell death in AML primary blasts sparing healthy blood cells **A.** AML primary blasts or **B.** mature normal blood cells were treated at different concentrations of emetine (1.5, 15, 150 nM). Cell viability was analyzed by flow cytometry at day 1 (left panel) and 3 (right panel) after treatment. Each symbol represents an AML patient sample as specified in the legend. B cells, myeloid cells and T cells were identified by the expression of CD19, CD13 and CD3 markers respectively. **C.** Primary AML patient samples were treated for 24 h with 150 nM emetine and cell viability was measured by flow cytometry. Each symbol represents an AML patient sample (summarized in Supplementary Table 3). Green, favorable and intermediate cytogenetics; red, unfavorable cytogenetics. * *p* < 0.05; ** *p* < 0.01; *** *p* < 0.001; ****p < 0.0001.

To further investigate the anti-leukemia effect of emetine, primary patient AML samples from different representative AML subtypes were tested against increasing concentrations of emetine for 1 and 3 days *ex vivo*. As shown for AML cell lines, primary AML samples were sensitive to emetine treatment at the nanoMolar range (Figure 3A). An optimal anti-leukemic drug for clinical practice should target leukemic blasts without significantly affecting normal blood cells to avoid unwanted side effects. Thus, mature blood cells from healthy donors were treated with emetine in the exact same conditions, as previously described with primary AML samples. A negligible cytotoxic effect was observed in B, T and myeloid cells when emetine was present in the culture medium (Figure 3B). Similarly, no effect was detected in human bone marrow hematopoietic cells treated as described above (Supplementary Figure 4). Taken together, emetine exerted a potent anti-leukemia effect on primary AML patient samples from all subtypes sparing normal blood cells, suggesting that emetine affects selectively AML blasts without targeting normal progenitor cells.

Due to the heterogeneous response observed upon emetine treatment at day 1, sensitivity to emetine was analyzed according to the prognostic cytogenetic category of AML samples (Figure 3C) based on diagnostic karyotype [19]. Remarkably, the unfavorable risk group responded better to emetine treatment than favorable/intermediate risk group as shown in Figure 3C.

**Figure 4:**
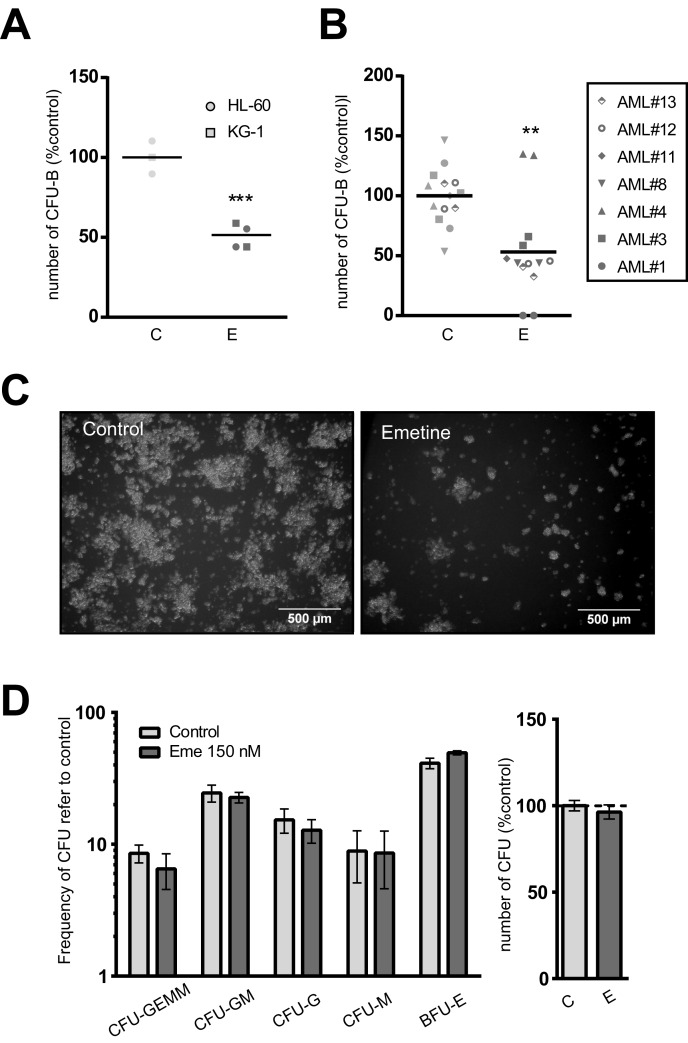
Emetine differentially reduced the clonogenic capacity of AML cells without affecting hematopoietic stem cell function **A.** AML cell lines or **B.** AML primary blasts were treated with 150 nM emetine (E, dark grey) or vehicle control (C, light grey) for 18 h. Colonies were screened at day 5 (HL-60), 7 (KG-1) or 14 (primary AML blasts). Each symbol represents a cell line or an AML primary sample. Results are normalized to control. **C.** Representative light microscope images of colonies in control- (left panel) and emetine-treated (right panel) HL-60 cells. **D.** Lineage-depleted umbilical cord blood cells were treated with emetine 150 nM (E, dark grey) or vehicle control (C, light grey) for 18 h. Colonies were screened at day 14 based on morphological and cellularity criteria. Left panel shows the frequency of each colony subtype relative to control, while right panel represents the total number of CFUs relative to control. Bars represent the mean value of two cord blood samples and error bars represent SEM. ** *p* < 0.01; *** *p* < 0.001.

The balance between self-renewal and differentiation is tightly regulated in LSCs. Induction of terminal differentiation results in the abrogation of the self-renewal capacity [8]. Self-renewal potential can be measured *in vitro* and *ex vivo* by analyzing the clonogenic capacity in a semi-solid culture medium in the presence of instructive cytokines. AML cell lines (Figure 4A) and primary patient AML samples (Figure 4B) were treated with emetine for 18h and their ability to generate blast colonies was determined morphologically. Emetine treatment reduced the clonogenic capacity of AML cells by 50%. Not only the number of colonies formed decreased upon treatment, but also the cellularity of each colony was reduced (Figure 4C), suggesting that emetine treatment reduced the number of colony-initiating cells but also impaired their capacity to generate cells within the colony.

Similarly to healthy mature blood cells, lineage-depleted umbilical cord blood cells were treated with emetine for 18h. No significant differences were observed in the total number of colonies or in the relative frequency of each colony generated between vehicle-treated and drug-treated samples, confirming the lack of myelotoxicity on normal CB progenitors (Figure 4D).

**Figure 5:**
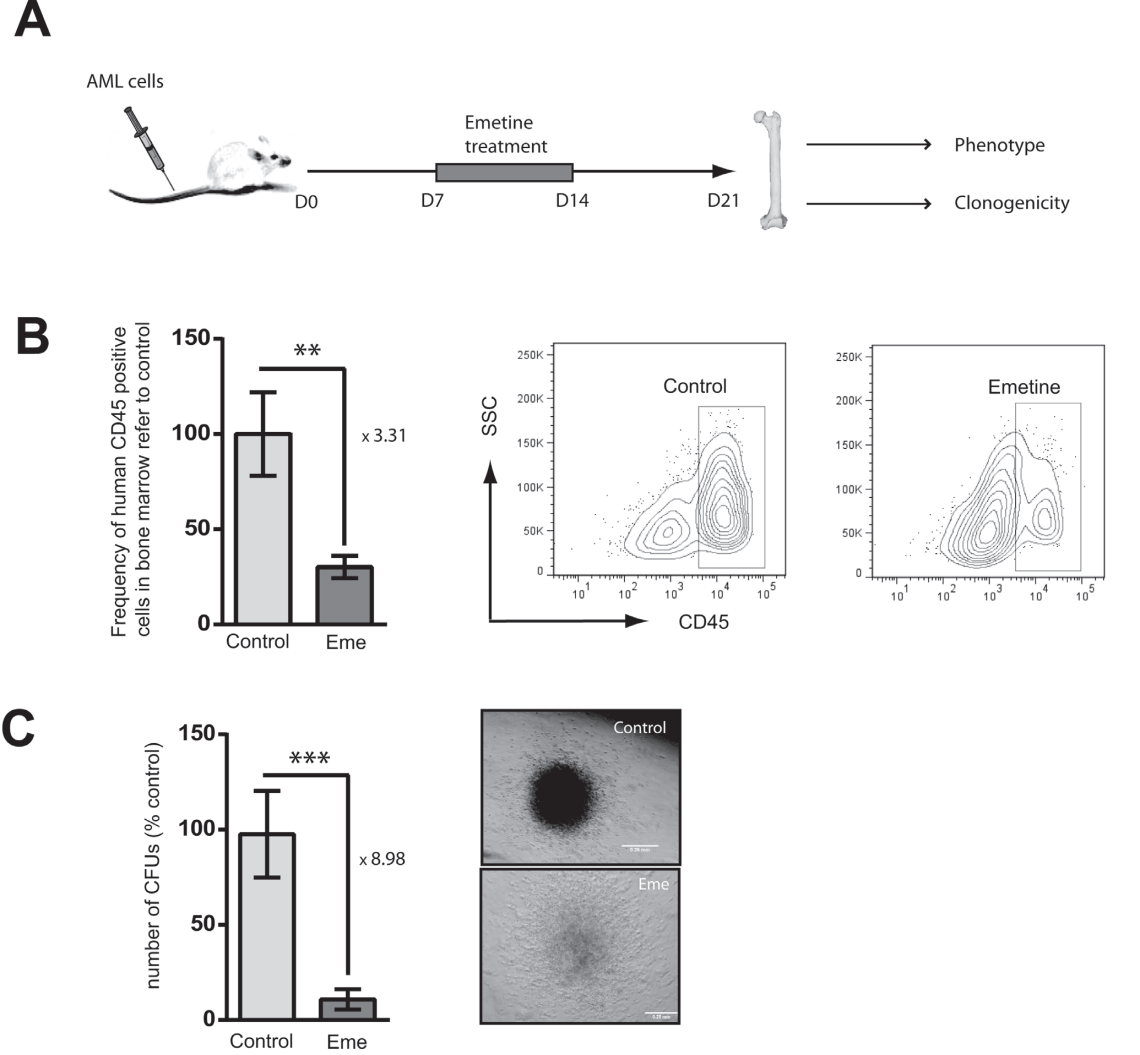
Emetine reduced AML burden in an *in vivo* xenotransplantation mouse model **A.** Conditioned NSG mice were intravenously (IV) transplanted with HL-60 cells. At day 7, mice were treated daily intraperitoneally (IP) with emetine (1 mg/kg) or vehicle control for 7 days. At day 21, bones were harvested and analyzed. **B.** Frequency of human CD45 positive cells in bone marrow referred to control (left panel) as assessed by flow cytometry. The right panel shows a representative flow cytometry plot of control- and emetine-treated bone marrow. **C.** Total number of *ex vivo* CFUs in control- and emetine-treated mice (left panel). Representative image of control- and emetine-treated colony. Error bars represent SEM. *p < 0.05.

In order to confirm the anti-leukemia activity of emetine, AML-bearing immunodeficient NSG mice were treated daily with the vehicle control or emetine (1 mg/kg weight) for 7 days, starting 7 days after transplantation when the leukemia was already established in the recipient mouse. At day 21, mice were analyzed for the presence of human AML cells in the bone marrow. Mice were left untreated 7 days after finishing the treatment regimen to allow AML cells to regenerate the disease. This treatment strategy was chosen to mimic clinic presentation of the disease (Figure 5A). A significant reduction in the frequency of human AML cells in bone marrow was observed in emetine-treated mice compared to vehicle-control treated mice (Figure 5B). Since the presence of human AML cells in bone marrow was severely reduced in emetine-treated mice (Figure 5B) and the analysis was performed 7 days after finishing the treatment to allow the remaining cells reinitiate the disease, the human leukemia residual clonogenic potential was evaluated *ex vivo*. In concordance with *in vitro* and *in vivo* data, a reduction in the number of colonies formed *ex vivo* was observed in human leukemia samples from emetine-treated bone marrow cells compared to control-treated animals (Figure 5C). In fact, the colonies generated from emetine-treated engrafted human leukemia bone marrow cells displayed a reduced cellularity as compared to control samples (Figure 5C). Taken together, emetine reduced both leukemia burden *in vivo* and the clonogenic capacity of leukemic cells upon treatment.

## DISCUSSION

Although the majority of AML patients respond initially to induction therapy and enter remission, more than half of these patients will experience a relapse episode, highlighting the necessity of new novel agents [2]. Here, an *in silico* screening was performed searching for AML differentiation-inducing FDA-approved small bioactive molecules. Emetine was identified and validated in AML cell lines. Treatment with emetine induced apoptosis of AML cell lines and primary samples at the nanomolar range. The presence of emetine decreased the clonogenic capacity of AML *ex vivo*. The anti-leukemic effect of emetine was even more pronounced in AML samples harboring high-risk cytogenetics. Moreover, emetine was able to reduce AML burden in a xenotransplantation mouse model and even more the clonogenic capacity. Taken together, our results support further clinical studies of emetine as an anti-leukemia novel drug in combinatory therapies and highlight the importance of HIF-1α, emetine molecular target, in drug resistance.

Emetine is a crystalline alkaloid derived from the ipecac root approved for the treatment of amoebiasis [20], although it is considered a second-line agent due to its toxicity associated with chronic usage [21, 22]. Emetine therapeutic dose for amoebiasis treatment ranges from 1-10 mg/kg for up to 10 days. Despite good cure rates, its use is today restricted by the need of parenteral (intramuscular or subcutaneous) administration and the occurrence of adverse reactions including cardio- and hepatotoxicity. Remarkably, significant toxicity is not experienced with a dose of 1 mg/kg daily subcutaneously [23]. With the emetine dose used in the *in vivo* experiments shown here, of 1 mg/kg per day for 7 days, no significant side effects were observed in treated mice compared to the vehicle control group. According to the “*Guidance for Industry: Estimating the Maximum Safe Starting Dose in Initial Clinical Trials for Therapeutics in Adult Healthy Volunteers”* published by the FDA 2005, the regimen used in the xenotransplantation mouse model corresponds to a 0.08 mg/kg dose in humans. Therefore, the anti-leukemic effect of emetine treatment described in this report is achieved at a concentration below toxicity.

AML is characterized by a differentiation blockage together with an increase in proliferation capacity. Differentiation therapies aimed to force terminal differentiation of AML cells that will convert AML cells into non-dividing growth-arrested cells, more sensitive to chemotherapeutics and with a reduced life span are promising. The ideal drug in this context would induce simultaneously or sequentially differentiation and apoptosis [23]. As shown here, emetine treatment induced apoptosis and partially differentiation, although the latter was only detected at a gene expression regulation level. The transcriptional activation of genes involved in myeloid differentiation and the loss of clonogenic capacity and, therefore, the lack of self-renewal activity upon emetine treatment, indicate that the differentiation program is activated. Additionally, apoptosis is detected after treatment both *ex vivo* and *in vitro*. Accordingly, emetine acts a bifunctional agent. Ideally, any therapy against AML should be aimed at inhibiting selectively the pivotal pathways of leukemogenesis without affecting normal hematopoietic cells, thus neutralizing leukemic cells and restoring normal hematopoiesis. Little effect on healthy mature peripheral and bone marrow blood cells was observed upon emetine treatment. Moreover, the clonogenic capacity of hematopoietic stem cells remained unaffected in the presence of emetine. Similarly, no bone marrow suppression has been reported in clinical trials where emetine has been evaluated [23], suggesting that the anti-leukemia effect of emetine is specific.

Although HIF-1α has been involved in hematopoietic malignancies, its specific role remains unclear. Several studies indicated that HIF-1α is elevated in LSCs and emerging evidence suggests that HIF-1α may be required for survival of LSCs [24]. At a clinical level, the expression of HIF-1α correlates with chemoresistance and poor prognosis in AML patients [25, 26]. Interestingly, AML samples harboring high-risk cytogenetics, associated to poor response to state-of-the-art AML chemotherapy, were more sensitive to emetine. Moreover, HIF-1α promotes quiescence of leukemic cells residing in the endosteal niches, mainly LSCs, and could contribute to the persistence of residual leukemic cells in AML [27]. Among all the targets described for emetine, in our system emetine seemed to decrease HIF-1α protein levels. In concordance with previous reports studying echinomycin [24], disruption of HIF-1α by emetine eliminated LSCs.

Due to the heterogeneity and dynamics of AML biology, a single agent used as monotherapy is unlikely to be capable to completely eradicate the disease. Induction of sensitivity to chemotherapy would allow the reduction of the effective concentration of chemotherapy and, potentially, decrease the severity of side effects. Emetine and ara-C synergized to induce cytotoxicity in AML cells. In fact, the combination of 1/2 of their EC50 reduced cell viability by 50%. Our results suggest that emetine constitutes an AML chemosensitizer that can eliminate LSC function, supporting further clinical investigation to elucidate its therapeutic potential.

In conclusion, this study validated the *in silico* predicted effect of emetine as a differentiating AML agent in AML cell lines, a group of diverse AML primary samples, and in a mouse AML-transplanted model. The anti-leukemic effect of emetine was shown in terms of induction of cytotoxicity and apoptosis, as well as significant reduction of clonogenic activity, suggesting targeting of LSCs. Moreover, the selective anti-leukemic effect of emetine together with the safety of the dose range required to exert this effect support the development of this agent in clinical practice.

## MATERIALS AND METHODS

### Identification of genes related to myeloid differentiation with connectivity maps

Gene signature associated with vitamin D3-induced differentiation in HL60 cells was obtained from GSE982 (GSM15451, GSM15452, GSM15453, GSM15576, GSM15577, GSM15578, GSM15561, GSM15562 and GSM15563). Raw files (.cel) were normalized and probe sets with a differential expression of at least 2-fold of change and *p* value < 0.005 were chosen using GenePattern software. The 500 top-ranking downregulated and 500 top-ranking upregulated probes during vitamin D3 treatment were selected for *in silico* signature-based screening Connectivity Maps (Supplementary Tables 1 and 2) [11].

### AML cell lines

AML cell lines HL-60 (ACC-3) [28], KG-1 (ACC-14) [29], MonoMac-1 (ACC-252) [30], Kasumi-1 (ACC-220) [31] and THP-1 (ACC-16) [32] were obtained from DSMZ (Germany). Cell lines were used within the first 6 months after resuscitation from reception or resuscitation from the DSMZ cell repository where they were genotypically characterized. Chemotherapy-resistant cells are HL-60 derived lines whose characteristics are summarized in Supplementary Figure 1.

### Primary samples

Primary AML samples were obtained from patients diagnosed with AML at Hospital Clínic of Barcelona (Spain). AML diagnosis and classification was based on accepted WHO criteria. Main AML patient's characteristics are summarized in Supplementary Table 3. Samples were obtained from bone marrow and mononuclear cells (MNCs) were isolated by Ficoll density gradient centrifugation (GE). All patients provided written informed consent in accordance with the Declaration of Helsinki, and the study was approved by the Ethics Committee of Hospital Clínic of Barcelona. Blood mature MNCs were isolated from healthy-donor buffy coats provided by Banc de Sang i Teixits (Barcelona, Spain). Umbilical cord blood MNCs were obtained after Ficoll density gradient centrifugation and were depleted for lineage marker-positive cells using magnetic beads (Milteny biotech).

### Drugs

Emetine dihydrochloride hydrate and Ara-C were obtained from Sigma-Aldrich.

### Cytotoxicity assay

7.5 × 10^5^ cells per mL were cultured in 96-well plates in complete medium. Emetine and Ara-C were added at indicated concentrations. Cell viability was measured by 7-AAD (eBioscience) exclusion and Hoechst33342 (Sigma) positivity staining by flow cytometry; and cell count was obtained by volume in a FACSCantoII cytometer (BD). Statistical analysis and EC50 determination were calculated in GraphPad (Prism software). FlowJo software (TriStar) was used for flow cytometry analysis.

### Apoptosis analysis

MonoMac-1 and Kasumi-1 cells were treated for 18h. Apoptosis was measured using the Annexin V apoptosis detection kit (BD) according to the manufacturer's instructions. Data were collected by flow cytometry (FACSCanto II, Becton-Dickinson) and analyzed in FlowJo software (Tristar).

### Cell cycle analysis

5 × 10^5^ cells per mL were cultured in 96-well plates in complete medium. 24h after treatment, cells were harvested, washed, and fixed and permeabilized in 70% ethanol (Sigma) at 4°C. DNA content was stained with 7-AAD (eBioscience) and measured by flow cytometry (FACSVerse and FACSCantoII, BD).

### Real time PCR

RNA was isolated using the Total RNA purification kit (Norgen biotek) according to the manufacturer's instructions and reverse transcriptase PCR was performed using the qScript cDNA synthesis kit (Quanta Biosciences). Quantitative PCR was performed using a Step One Plus Real-Time PCR System (Applied Biosystems) with a Power SYBR Green PCR mastermix (Applied Biosystems) according to the manufacturer's instructions and using specific primers for each gene (summarized in Supplementary Table 4). GAPDH expression was used as control.

### Synergy assessment

Synergism was assessed by Excess Over Bliss Additivism (EOBA) [18]. Fractional inhibition was obtained from cytotoxicity assays and EOBA was calculated for each concentration by subtracting the predicted Bliss additive effect - the addition of the fractional effect of each drug alone minus the product of both fractional effects [33] - to the fractional effect of drugs combined (EOBA = E_AB_ - E_A_ - E_B_ + E_A_· E_B_). Results are expressed in percentage.

### Clonogenicity assay

50 × 10^3^ primary AML cells or 1 × 10^3^ cells from AML cell lines were treated for 24 h with the indicated compound and mixed with 1 mL of MethoCult H4034 Optimum (StemCell Technologies). Colonies were screened based on morphology and cellularity at day 5 (HL-60), day 7 (KG-1) or day 14 (primary AML cells).

### Western blot

HL-60 and KG-1 cells (10 × 10^6^ cells) were incubated with the indicated compounds for 24 h. Cells were harvested and lysed in RIPA buffer. The protein extract was subjected to SDS-polyacrylamide gel (BioRad) electrophoresis and transferred to a nitrocellulose membrane (BioRad). Mouse anti-human HIF-1α and goat anti-β-actin antibodies were purchased from BD and Abcam respectively.

### Intracellular staining

Cells were fixed, permeabilized, and stained with anti-Phospho-Akt (Thr308) clone C31E5E from Cell Signaling Technologies following manufacturers' recommendations. RPE-coupled anti-rabbit secondary antibodies (Life Technologies) were used and samples were acquired in a FACSCanto II (BD).

### *In vivo* studies

NOD.Cg-Prkdc^scid^Il2rg^tm1Wjl^/SzJ (NSG, Jackson Laboratories) mice were myeloablated by busulfan (3 mg/kg IP) at day −1. At day 0, 10 million HL-60 cells were injected IV and left untreated for one week. From day 7 to day 14, 8 mice were treated daily with emetine (1 mg/kg IP), while the other 9 mice were treated with saline vehicle. At day 21, mice were sacrificed and their bones (iliac crests, femurs and tibias) were harvested. Cells for cytometric analysis were stained with Hoechst 33342 and PE-Cy7 Anti-Human CD45 (BD Pharmingen) and acquired in a FacsCantoII (BD) cytometer. Live cells were gated based on their size, complexity, absence of 7-AAD staining and the expression of human CD45 on the surface.

## SUPPLEMENTARY MATERIALS FIGURES AND TABLES







## References

[1] Burnett A, Wetzler M, Lowenberg B (2011). Therapeutic advances in acute myeloid leukemia. J Clin Oncol.

[2] Roboz GJ (2012). Current treatment of acute myeloid leukemia. Curr Opin Oncol.

[3] Lapidot T, Sirard C, Vormoor J, Murdoch B, Hoang T, Caceres-Cortes J, Minden M, Paterson B, Caligiuri MA, Dick JE (1994). A cell initiating human acute myeloid leukaemia after transplantation into SCID mice. Nature.

[4] Bonnet D, Dick JE (1997). Human acute myeloid leukemia is organized as a hierarchy that originates from a primitive hematopoietic cell. Nat Med.

[5] Becker MW, Jordan CT (2011). Leukemia stem cells in 2010: current understanding and future directions. Blood Rev.

[6] Dohner H, Estey EH, Amadori S, Appelbaum FR, Buchner T, Burnett AK, Dombret H, Fenaux P, Grimwade D, Larson RA, Lo-Coco F, Naoe T, Niederwieser D, Ossenkoppele GJ, Sanz MA, Sierra J (2010). Diagnosis and management of acute myeloid leukemia in adults: recommendations from an international expert panel, on behalf of the European LeukemiaNet. Blood.

[7] Nowak D, Stewart D, Koeffler HP (2009). Differentiation therapy of leukemia: 3 decades of development. Blood.

[8] Sachlos E, Risueno RM, Laronde S, Shapovalova Z, Lee JH, Russell J, Malig M, McNicol JD, Fiebig-Comyn A, Graham M, Levadoux-Martin M, Lee JB, Giacomelli AO, Hassell JA, Fischer-Russell D, Trus MR (2012). Identification of drugs including a dopamine receptor antagonist that selectively target cancer stem cells. Cell.

[9] Moreno-Martinez D, Nomdedeu M, Lara-Castillo MC, Etxabe A, Pratcorona M, Tesi N, Diaz-Beya M, Rozman M, Montserrat E, Urbano-Ispizua A, Esteve J, Risueno RM (2014). XIAP inhibitors induce differentiation and impair clonogenic capacity of acute myeloid leukemia stem cells. Oncotarget.

[10] McCarthy DM, San Miguel JF, Freake HC, Green PM, Zola H, Catovsky D, Goldman JM (1983). 1,25-dihydroxyvitamin D3 inhibits proliferation of human promyelocytic leukaemia (HL60) cells and induces monocyte-macrophage differentiation in HL60 and normal human bone marrow cells. Leuk Res.

[11] Lamb J, Crawford ED, Peck D, Modell JW, Blat IC, Wrobel MJ, Lerner J, Brunet JP, Subramanian A, Ross KN, Reich M, Hieronymus H, Wei G, Armstrong SA, Haggarty SJ, Clemons PA (2006). The Connectivity Map: using gene-expression signatures to connect small molecules, genes, and disease. Science.

[12] Pabst T, Mueller BU, Harakawa N, Schoch C, Haferlach T, Behre G, Hiddemann W, Zhang DE, Tenen DG (2001). AML1-ETO downregulates the granulocytic differentiation factor C/EBPalpha in t(8;21) myeloid leukemia. Nat Med.

[13] Kastner P, Chan S (2008). PU. 1: a crucial and versatile player in hematopoiesis and leukemia. Int J Biochem Cell Biol.

[14] Grollman AP (1966). Structural basis for inhibition of protein synthesis by emetine and cycloheximide based on an analogy between ipecac alkaloids and glutarimide antibiotics. Proc Natl Acad Sci U S A.

[15] Grollman AP (1968). Inhibitors of protein biosynthesis. V. Effects of emetine on protein and nucleic acid biosynthesis in HeLa cells. J Biol Chem.

[16] Schweighoffer T, Schweighoffer E, Apati A, Antoni F, Molnar G, Lapis K, Banfalvi G (1991). Cytometric analysis of DNA replication inhibited by emetine and cyclosporin A. Histochemistry.

[17] Zhou YD, Kim YP, Mohammed KA, Jones DK, Muhammad I, Dunbar DC, Nagle DG (2005). Terpenoid tetrahydroisoquinoline alkaloids emetine, klugine, and isocephaeline inhibit the activation of hypoxia-inducible factor-1 in breast tumor cells. J Nat Prod.

[18] Borisy AA, Elliott PJ, Hurst NW, Lee MS, Lehar J, Price ER, Serbedzija G, Zimmermann GR, Foley MA, Stockwell BR, Keith CT (2003). Systematic discovery of multicomponent therapeutics. Proc Natl Acad Sci U S A.

[19] Mrozek K, Heerema NA, Bloomfield CD (2004). Cytogenetics in acute leukemia. Blood Rev.

[20] Goodson JA, Goodwin LG, Gorvin JH, Goss MD, Kirby KS, Lock JA, Neal RA, Sharp TM, Solomon W (1948). The chemotherapy of amoebiasis; amines derived formally from emetine. Br J Pharmacol Chemother.

[21] Dempsey JJ, Salem HH (1966). An enzymatic electrocardiographic study on toxicity of dehydroemetine. Br Heart J.

[22] Goldsmith RS, Katzung BG (1998). Antiprotozoal Drugyzs in Basic and Clinical Pharmacology. Basic & Clinical Pharmacology.

[23] Mastrangelo MJ, Grage TB, Bellet RE, Weiss AJ (1973). A phase I study of emetine hydrochloride (NSC 33669) in solid tumors. Cancer.

[24] Wang Y, Liu Y, Malek SN, Zheng P (2011). Targeting HIF1alpha eliminates cancer stem cells in hematological malignancies. Cell Stem Cell.

[25] Song K, Li M, Xu XJ, Xuan L, Huang GN, Song XL, Liu QF (2014). HIF-1alpha and GLUT1 gene expression is associated with chemoresistance of acute myeloid leukemia. Asian Pac J Cancer Prev.

[26] Deeb G, Vaughan MM, McInnis I, Ford LA, Sait SN, Starostik P, Wetzler M, Mashtare T, Wang ES (2011). Hypoxia-inducible factor-1alpha protein expression is associated with poor survival in normal karyotype adult acute myeloid leukemia. Leuk Res.

[27] Matsunaga T, Imataki O, Torii E, Kameda T, Shide K, Shimoda H, Kamiunten A, Sekine M, Taniguchi Y, Yamamoto S, Hidaka T, Katayose K, Kubuki Y, Dobashi H, Bandoh S, Ohnishi H (2012). Elevated HIF-1alpha expression of acute myelogenous leukemia stem cells in the endosteal hypoxic zone may be a cause of minimal residual disease in bone marrow after chemotherapy. Leuk Res.

[28] Gallagher R, Collins S, Trujillo J, McCredie K, Ahearn M, Tsai S, Metzgar R, Aulakh G, Ting R, Ruscetti F, Gallo R (1979). Characterization of the continuous, differentiating myeloid cell line (HL-60) from a patient with acute promyelocytic leukemia. Blood.

[29] Koeffler HP, Golde DW (1978). Acute myelogenous leukemia: a human cell line responsive to colony-stimulating activity. Science.

[30] Steube KG, Teepe D, Meyer C, Zaborski M, Drexler HG (1997). A model system in haematology and immunology: the human monocytic cell line MONO-MAC-1. Leuk Res.

[31] Asou H, Tashiro S, Hamamoto K, Otsuji A, Kita K, Kamada N (1991). Establishment of a human acute myeloid leukemia cell line (Kasumi-1) with 8;21 chromosome translocation. Blood.

[32] Tsuchiya S, Yamabe M, Yamaguchi Y, Kobayashi Y, Konno T, Tada K (1980). Establishment and characterization of a human acute monocytic leukemia cell line (THP-1). Int J Cancer.

[33] Berenbaum MC (1981). Criteria for analyzing interactions between biologically active agents. Adv Cancer Res.

[34] Piret JP, Mottet D, Raes M, Michiels C (2002). CoCl2, a chemical inducer of hypoxia-inducible factor-1, and hypoxia reduce apoptotic cell death in hepatoma cell line HepG2. Ann N Y Acad Sci.

