# Supplementary material for: Emetine induces chemosensitivity and reduces clonogenicity of acute myeloid leukemia cells
Source: Oncotarget. 2016 Mar 15;7(17):23239–50. doi: 10.18632/oncotarget.8096 (PMC5029623; doi:10.18632/oncotarget.8096)
Supplement: Supplementary file 1 [file oncotarget-07-23239-s001.pdf]

Emetine induces chemosensitivity and reduces clonogenicity of acute myeloid leukemia cells

Supplementary Material

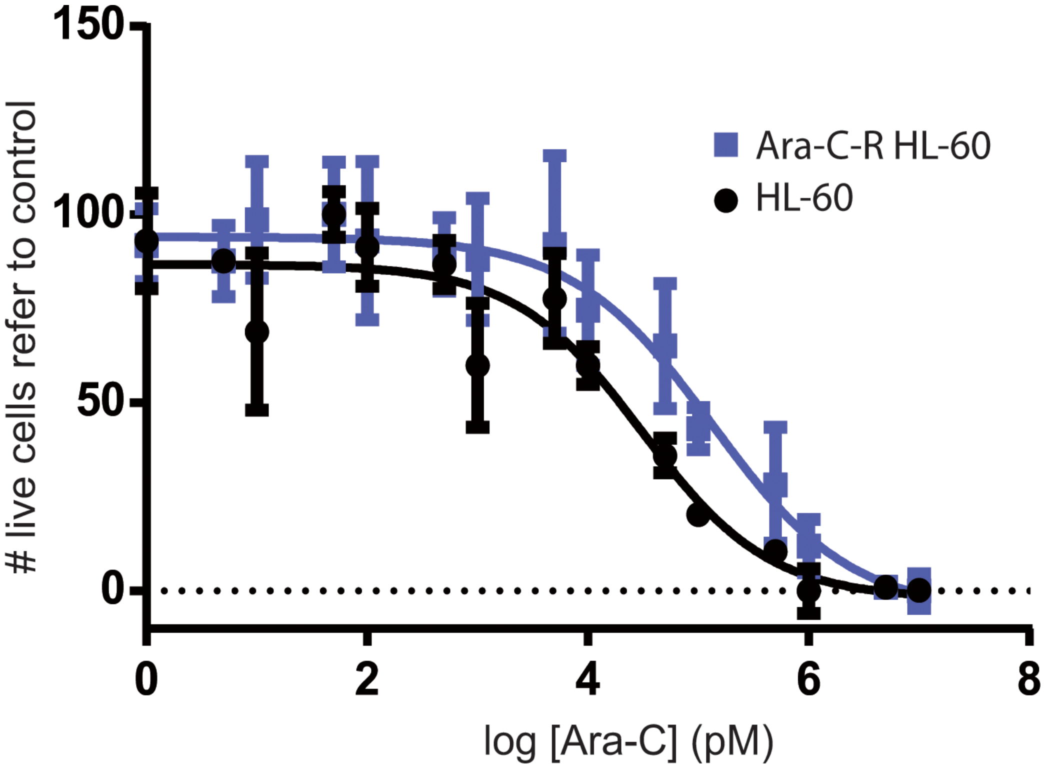

Cell line	EC50 (nM)
Ara-C-R HL-60	133
HL-60	31

Supplementary Figure 1. HL-60 cells resistant to chemotherapeutics. Cell viability at different Ara-C doses (pM) for 24 h. Blue, Ara-C resistant cells; black, parental cell line.

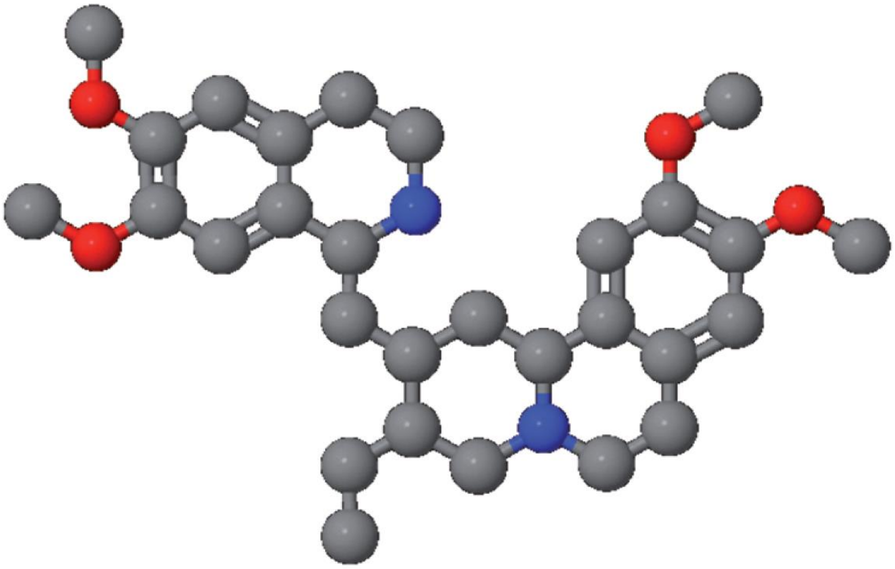

Emetine

Supplementary Figure 2. Emetine structure. Chemical structure of Emetine.

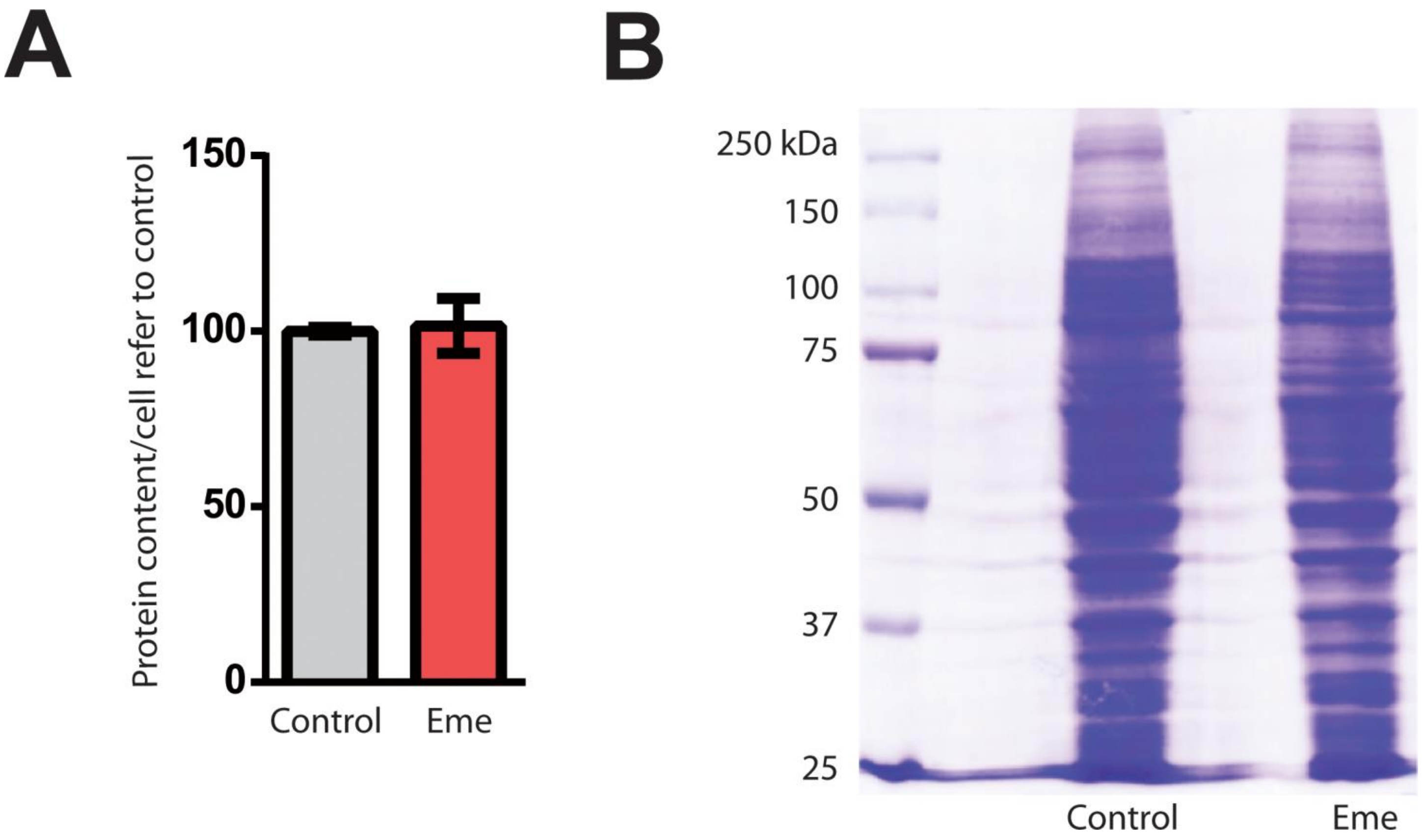

Supplementary Figure 3. Emetine treatment does not affect the protein profile in HL-60 cells. HL-60 cells were treated with Emetine and proteins were extracted. **A**. Protein concentration measured by BCA is represented. Bars correspond to the mean value of triplicates and error bars correspond to SEM. **B**. Coomassie-staining profile of control and emetine-treated cells. A representative gel is presented.

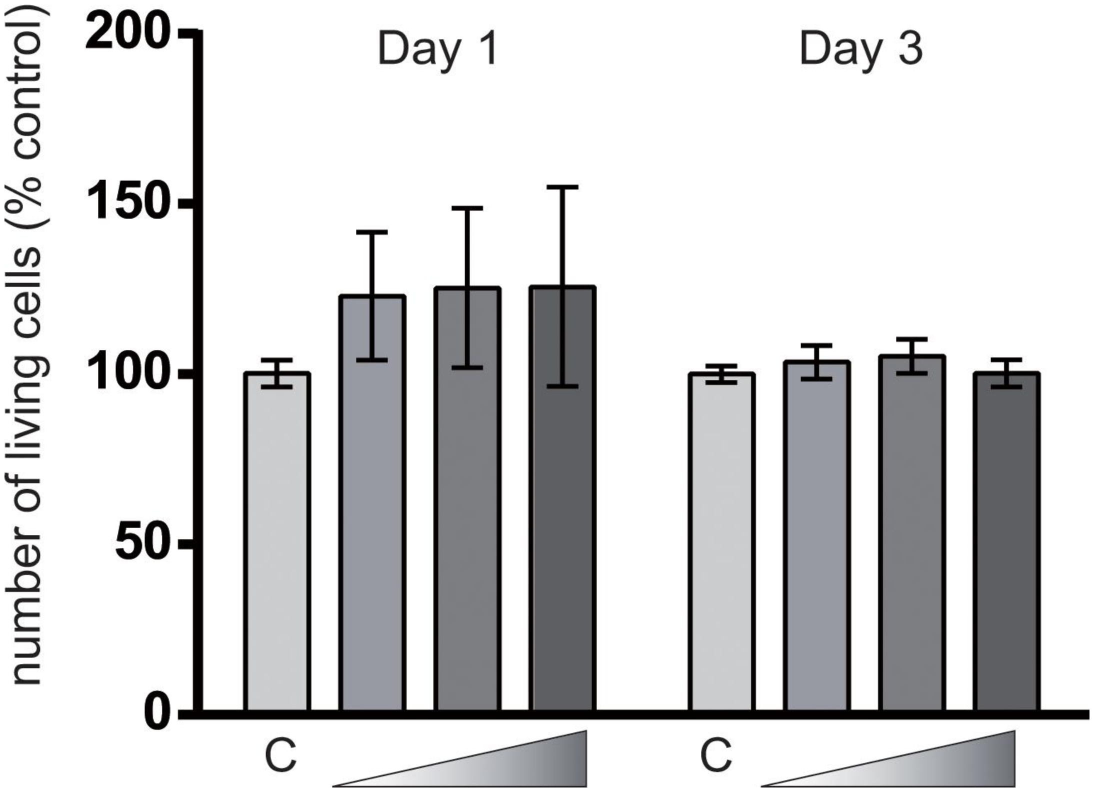

Supplementary Figure 4. Emetine treatment spares healthy bone marrow cells. Mature normal bone marrow cells were treated at different concentrations of emetine (1.5, 15, 150 nM). Cell viability was analyzed by flow cytometry at day 1 and 3 after treatment.

Supplementary Table 1. Genes upregulated during vitamin D3-induced differentiation.

Supplementary Table 2. Genes downregulated during vitamin D3-induced differentiation.

Supplementary Table 3. AML patients' characteristics.

AML sample	Gender	Age (yr)	WHO subtype	WBC count#	% Blasts in PB	% Blasts in BM	Karyotype	Additional molecular features
#1	M	28	AML without maturation	143	98	90	46, XY	FLT3-ITD
#2	M	40	AML without maturation	52	66	80	46,XY	Biallelic CEBPA mutation
3	F	34	AML with myelodysplasia-related changes	32	16	44	45,XX, -7	$FLT3^{wt}$ NPM^{wt}
#4	M	45	AML with t(6,9)	40	58	43	46,XY,t(6;9)(p23;q34)	FLT3-ITD
#5	M	48	AML with t(8;21)(q22;q22)	54	5	24	46,XY,t(8;21)(q22;q22)	NPM^{wt} FLT3 N/A
#6	M	61	AML with t(8;21)(q22;q22)	21.4	51	89	45,X-Y,t(8;21)(q22;q22)/46,XY	$FLT3^{wt}$ NPM^{wt}
#7	F	58	AML with myelodysplasia-related changes	100.7	45	80	46,XX,del(5)(q23q33), t(8;9)(p11;q34)	$FLT3^{wt}$ NPM^{wt}
#8	M	24	AML with myelodysplasia-related changes	7.1	83	30	46,XY	$FLT3^{wt}$ NPM^{wt}
#9	M	49	AML with myelodysplasia-related changes	76.4	42	26	46-47,XY,del(5)(q22q34),del(6)(q22q25),del(7)(q22q23),-8,-9,add(11)(q23),+i(11)(q11),-16,+mar1,+mar2,+mar3[cp8].	$FLT3^{wt}$ NPM^{wt}
#10	M	22	AML with t(8;21)(q22;q22);	20.4	83	69	45,X,-Y,t(8;21)(q22;q22)/46,XY	FLT3 ITD
#11	F	60	AML with myelodysplasia-related changes	218.1	68	36	48,XX,+8,+21	$FLT3^{wt}$ NPM^{wt}
#12	M	63	AML with myelodysplasia-related changes	55	84	21	46,XY	FLT3 ITD
#13	M	61	AML with mutated NPM1	N/A	33	72	46,XY	NPM1 mut DNMT3A mut

Supplementary Table 4. Primer sequences.

Primer	Sequence
GAPDH forward	GTGGACCTGACCTGCCGTCT
GAPDH reverse	GGAGGAGTGGGTGTCGCTGT
PU.1 forward	GGGGTGGAAGTCCCAGTAAT
PU.1 reverse	ACGGATCTATACCAACGCCA
C/EBPα forward	TTGCTGCCTTCCTTTCCTACTGC
C/EBPα reverse	GCTGGAGAGGATGGACTTACTTGA
ABCB1 forward	CCCATCATTGCAATAGCAGG
ABCB1 reverse	TGTTCAAACTTCTGCTCCTGA
Bcl-X forward	GAGGCAGGCGACGAGTTTGAA
Bcl-X reverse	TGGGAGGGTAGAGTGGATGGT
